# Effects of the surface physico-chemical properties and the surface textures on the initial colonization and the attached growth in algal biofilm

**DOI:** 10.1186/s13068-016-0451-z

**Published:** 2016-02-16

**Authors:** Martin Gross, Xuefei Zhao, Vernon Mascarenhas, Zhiyou Wen

**Affiliations:** Department of Food Science and Human Nutrition, 2312 Food Science Building, Iowa State University, Ames, IA 50011 USA; Department of Agricultural and Biosystems Engineering, Iowa State University, Ames, IA 50011 USA; Department of Chemical Biological Engineering, Iowa State University, Ames, IA 50011 USA

**Keywords:** Algae attachment, Attached growth, Surface texture, Biofilm, Contact angle

## Abstract

**Background:**

Algal biofilm reactors represent a promising cultivation system that can economically produce biomass without the need for expensive harvesting operations. A critical component of algal biofilm systems is the material used for attachment. This research reports a comprehensive study of the effects of material surface physico-chemical properties, the surface texture, and their interactions on the initial colonization and the long-term attached growth in algal biofilm systems. A total of 28 materials with a smooth surface were tested for initial cell colonization and it was found that the tetradecane contact angle of the materials had a good correlation with cell attachment. The effects of surface texture were evaluated using mesh materials (nylon, polypropylene, high-density polyethylene, polyester, aluminum, and stainless steel) with openings ranging from 0.05 to 6.40 mm.

**Results:**

The mesh materials with an opening of 0.5 mm resulted in the highest attachment. The interaction of surface physico-chemical properties and surface texture, and their co-effects on the cell attachment, was quantitatively described using a second-order polynomial regression. The long-term algal attached growth for the different materials showed a trend similar to that found in initial colonization.

**Conclusions:**

Collectively, nylon and polypropylene mesh with 0.50–1.25 mm openings resulted in the best initial colonization and long-term attached growth, with a 28–30 g m^−2^ biomass yield and 4.0–4.3 g m^−2^ day biomass productivity being achieved on a pilot-scale revolving algal biofilm system.

## Background

Microalgae have been researched for production of a variety of fuels, feeds, and chemicals. It also has been used to mitigate various pollutants found in municipal wastewater [1, 2] agricultural effluents [3], and animal housing air with high levels of ammonia and CO_2_ [4]. Current cultivation systems such as raceway ponds and photobioreactors require costly and energy intensive methods to harvest the suspended microscopic algae cells from liquid. For example, Davis et al. [5] reported that harvesting alone contributes 21 % of capital costs in an open-pond system.

Biofilm-based algal culture systems have proven effective in reducing expensive algae harvesting operations [6]. In biofilm systems, algae are attached on the surface of a material and are easily harvested via scraping. The harvested biomass has a water content similar to post-centrifuged biomass (80–90 % moisture) [7–9]. In addition to the benefit of easy harvesting, algal biofilms also have the features of minimizing light limitation and enhancing CO_2_ mass transfer. The solids retention time of the cells is also increased due to the separation of the algal cells and liquid medium, which is a benefit in water treatment applications [6].

Various algal biofilm reactors have been developed in past decades and have drawn renewed interest in recent years. A comprehensive review of biofilm-based algal cultivation systems, including the reactor design configurations, the pros and cons of the systems, and the factors affecting biofilm growth performance has been provided in a recent review [6]. In terms of the mechanism of algae-material attachment, various theories and hypotheses have been proposed such as hydrophobic interactions [10], acid–base interactions [11, 12], and surface energy [13]. The effect of material on the algal attachment and attached growth is a rather complicated process. On one hand, the material surface physico-chemical properties such as hydrophobicity [14], surface energy [13], and dispersive surface energy [15] play an important role for the initial algal colonization, but this highly depends on the materials and model strain used. On the other hand, the different micro-patterns (texture) of attachment material surface affect cell recruitment and retention as well. The materials with an appropriate surface texture provide a “shelter” for the attached cells; as a result, the sloughing of the attached cells can be significantly reduced. Indeed, it has been reported that altering the surface can drastically increase algal attachment [16–19].

It should be noted that most of the previous studies focused on initial colonization of the algal cells to the fresh material surface. Once the colony is established, the long-term sustained attached growth on the material is rarely reported. In addition, the other surface physico-chemical properties and surface texture of the materials have been reported individually; the interactions between these two properties and their combined effect on the cell attachment and growth have not been well understood.

Selection of an appropriate attachment material is important in the development of an algal biofilm system. However, only a limited number of materials have been studied. In this work, a comprehensive study of 28 smooth materials with different surface physico-chemical properties and 6 textured materials with various levels of surface texture were investigated for their roles in cell attachment and growth, with the aim to provide insight into the cell attachment mechanism. Additionally, this work will assist in determining a promising attachment material that can be implemented for commercial algal biofilm growth systems.

## Results

### Algal attachment as a function of material surface physico-chemical properties

The surface physico-chemical properties, including water, glycerol, and tetradecane contact angle, as well as the free surface energy of the materials, were analyzed. As shown in Table 1, the liquid contact angle varied widely among different materials, with the surface energies, except aluminum and brass, in the range of the 20–60 mJ m^−2^. The data were consistent with the previous reports [20].

**Table 1:** Attachment materials (with smooth surface) and their surface physico-chemical properties using cell attachment tests

Attachment material	Liquid contact angle (°)			Surface energy (mJ m^−2^)
	Water	Glycerol	Tetradecane^a^	
Metals				
Aluminum	96.6	89.6	<2	168.0
Brass	89.4	94.2	<2	193.0
Stainless steel	80.9	80.3	<2	43.1
Plastics				
Acrylonitrile butadiene styrene (ABS)	84.7	78.2	23.4	36.7
Nylon	59.6	45.2	15.5	43.2
Polyethylene terephthalate (PETG)	68.5	69.0	<2	44.0
Chlorinated polyvinyl chloride (CPVC)	78.6	80.0	11.9	40.5
Delrin acetal resin	72.1	68.3	9.3	42.5
Polyester	82.7	74.2	6.3	41.0
Polylactic acid (PLA)	80.8	88.1	24.6	38.7
Polycarbonate	86.1	77.6	<2	46.0
Extruded acrylic	76.8	61.7	6.2	44.7
Extruded nylon	72.7	57.0	9.1	45.8
High-density polyethylene (HDPE)	88.0	73.9	3.0	35.3
Polypropylene	91.5	81.0	16.6	32.6
Polystyrene	75.8	76.8	<2	34.0
Low density polyethylene (LDPE)	89.1	60.4	<2	33.6
Polyvinyl chloride (PVC)	88.3	77.5	<2	33.0
Rexolite polystyrene	51.8	140.3	<2	38.3
Ultra-high-molecular-weight polyethylene	84.2	63.1	<2	37.2
Rubbers				
Buna-N rubber	38.2	92.0	30.6	31.4
Neoprene rubber	92.6	92.1	24.1	39.8
NORYL PPO	80.2	83.4	3.9	52.5
Gum rubber	59.6	81.0	26.9	31.8
Butyl rubber	92.9	93.0	33.8	29.9
Ethylene propylene diene monomer (EPDM)	110.4	85.2	32.1	34.9
Epichlorohydrin (ECH) rubber	55.5	58.6	<2	43.2
Hypalon rubber	71.2	83.5	16.6	57.9
Latex rubber	92.9	124.2	28.2	36.3
Polyurethane	89.8	83.1	<2	37.5
Santoprene rubber	89.8	95.8	43.5	26.1
Styrene-butadiene (SBR) rubber	84.2	89.0	23.8	44.9
Silicone rubber	58.2	85.2	12.3	21.3

^a^Tetradecane contact angles with <2° were below the measurement limit

**Fig. 1:**
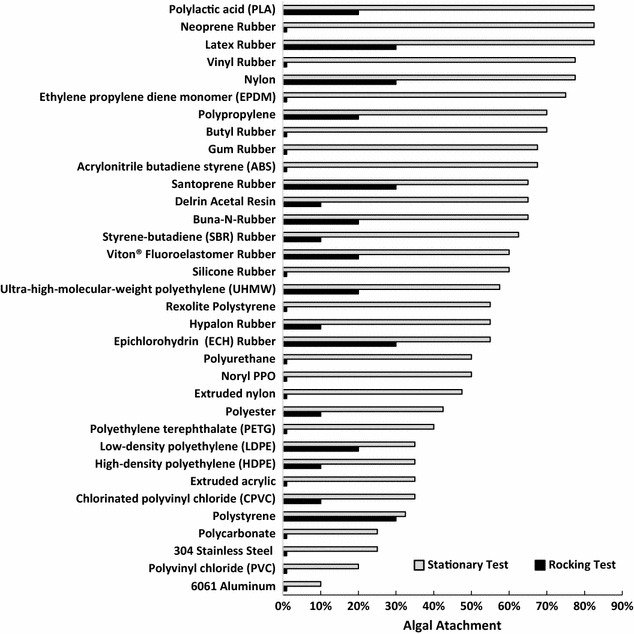
Algal colonization (represented as area covered by attached algae as a % of material surface) on different materials with smooth surface

Once the material surface physico-chemical properties were determined, the attachment of algal cells to the materials was determined. As shown in Fig. 1, polylactic acid, neoprene, and latex demonstrated the best attachment in stationary conditions. The cell attachment in the rocking condition, however, did not show the same trend and no clear relationship was found between the stationary and rocking tests.

Cell attachment was further correlated with the contact angles and surface energy in order to provide a quantitative relationship between cell attachment and surface physico-chemical properties. Since the surface energy calculation involves three liquid contact angles (water-, glycerol-, and tetradecane-based) (see “Methods” section), the correlation was performed with each of these contact angles. No obvious quantitative correlations were observed between cell attachment and surface energy (*R*^2^ = 0.03), water contact angle (*R*^2^ = 0.03), and glycerol contact angle (*R*^2^ = 0.06). However, a relatively strong correlation (*R*^2^ = 0.68) between algal attachment and tetradecane contact angle was observed.

### Algal attachment as a function of materials surface texture

Six materials were used to test the effects of surface texture on algal attachment. The criteria for selecting the materials were based on (1) coverage of a wide range of attachment performance, and (2) commercial availability without additional cost of custom fabricating. The capability of being flexible is another criterion in the material selection, as we envision the future implementation of the attachment materials will be in the revolving algal biofilm (RAB) system [8, 9]. Based on those criteria, two metals (aluminum and stainless steel) and four plastics (polyester, high-density polyethylene, nylon, and polypropylene) representing the good, modest, and poor attachment observed from the smooth surface test (Fig. 1) were selected. The materials between polypropylene and polyester (Fig. 1) were not selected because the special surface texture of those materials was not readily available from existing commercial sources. Polystyrene was also not selected due to the inflexibility of this material, although the attachment of this material in the rocking test was very good as well as in a previous study.

**Fig. 2:**
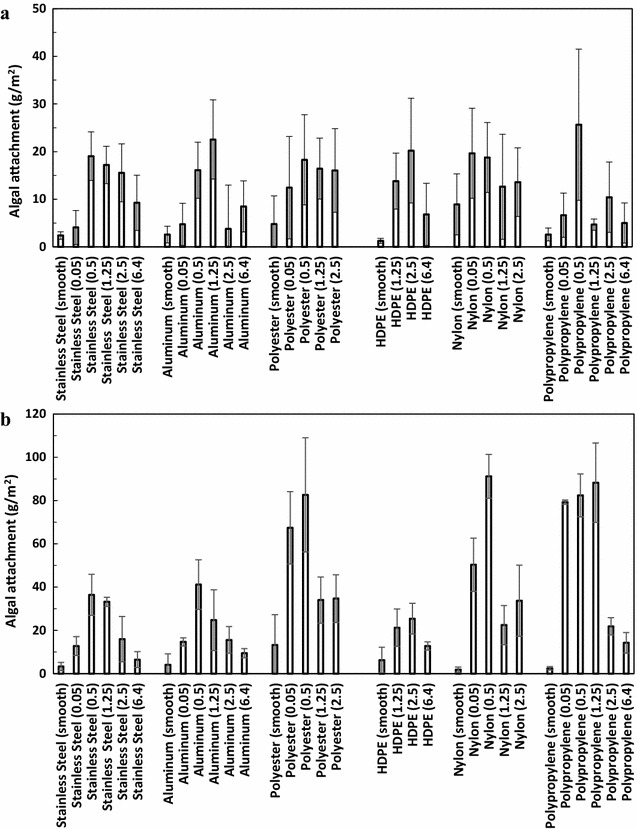
Algal attachment on materials with different textures at **a** stationary conditions and **b** rocking conditions. The *figure in the parenthesis* is the mesh opening size, mm

As shown in Fig. 2a, under the stationary conditions, the best algal attachment is difficult to determine due to the large errors observed, although the smooth surface and smallest pore size (0.05 mm) of the material (except HDPE) appeared to have the lowest level of attachment. Under the rocking conditions (Fig. 2b), the best algal attachment occurred on a mesh with a 0.5-mm opening for stainless steel, aluminum, polyester, and nylon. The HDPE mesh with a 0.5-mm opening was not available, and the best attachment was obtained with a 1.25–2.5-mm opening. For polypropylene, the mesh with 0.05, 0.5, and 1.25 mm openings resulted in similar attachment (Fig. 2b).

Following the quantitative determination of cell attachment, the effect of surface texture on cell attachment (under rocking conditions) was further evaluated using scanning electron microscopy (SEM) observation. Two materials (nylon and aluminum) were selected for SEM observation because nylon resulted in good attachment and aluminum resulted in poor attachment (Fig. 2b). It should be noted that polyester and polypropylene had a similar attachment performance as nylon. However, compared to polyester and polypropylene, the cell attachment in nylon varied more widely with different opening sizes, which is an ideal feature to reveal the cell attachment difference among different mesh openings.

**Fig. 3:**
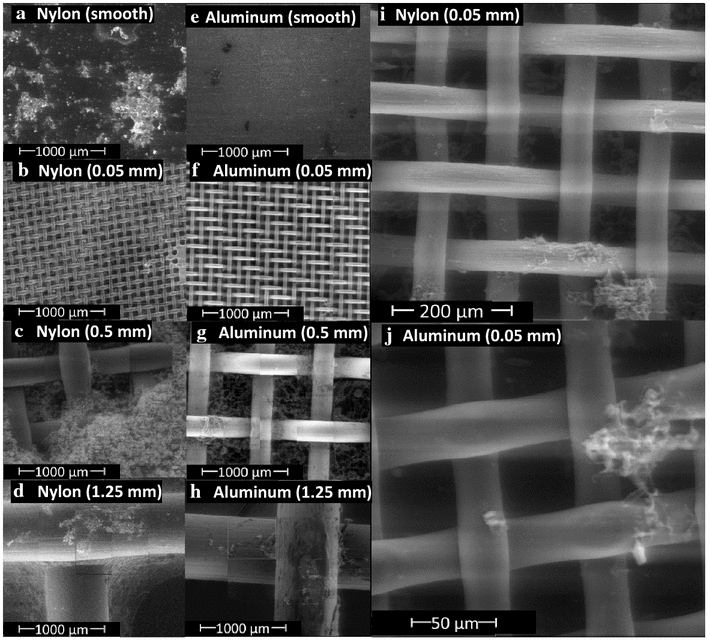
SEM images of algal attachment on nylon and aluminum with different mesh size openings. **a** Nylon with smooth surface, **b** nylon mesh with 0.05 mm opening, **c** nylon mesh with 0.05 mm opening, **d** nylon mesh with 1.25 mm opening, **e** aluminum with smooth surface, **f** aluminum mesh with 0.05 mm opening, **g**, aluminum mesh with 0.5 mm opening, **h** aluminum mesh with 1.25 mm opening, **i** nylon mesh with 0.05 mm opening with increased magnification, and **j** aluminum mesh with 0.05 mm mesh with increased magnification. The experiments were tested under rocking conditions

The images in Fig. 3 are typical results representing the cell attachment. First, cell attachment on the same material with different sizes of mesh openings was compared at 50× magnification (Fig. 3a–d for nylon; and Fig. 3e–h for aluminum). It shows that the mesh with 0.5 mm opening attached the most cells (Fig. 3c for nylon, 3 g for aluminum). Such qualitative observation is in agreement with the quantitative cell attachment results shown in Fig. 2b. Second, the cell attachment on the different materials were compared with the same mesh opening (Fig. 3a vs e, 3b vs f; 3c vs g, and 3d vs h), it clearly indicates that nylon attached more cells than the aluminum under the same mesh size. Third, the SEM image magnification was further increased to provide a clear picture of the cell attachment. As shown in Fig. 3i, j which were magnified 200× and 500×, respectively, the algal cell aggregates formed can be clearly seen to exceed the 0.05 mesh pore size. It is believed that the inability of algal aggregates to fit into the mesh pores could be the reason for decreased cell attachment at the 0.05 mm mesh opening (Fig. 2b).

### Co-effect of surface physico-chemical properties and texture on algal attachment

The previous results show that the cell attachment varied with surface physico-chemical properties and the surface texture of the attachment material. In this section, the interactions and combined effects of these two parameters on the cell attachment were evaluated through a series of statistically based analyses.

**Fig. 4:**
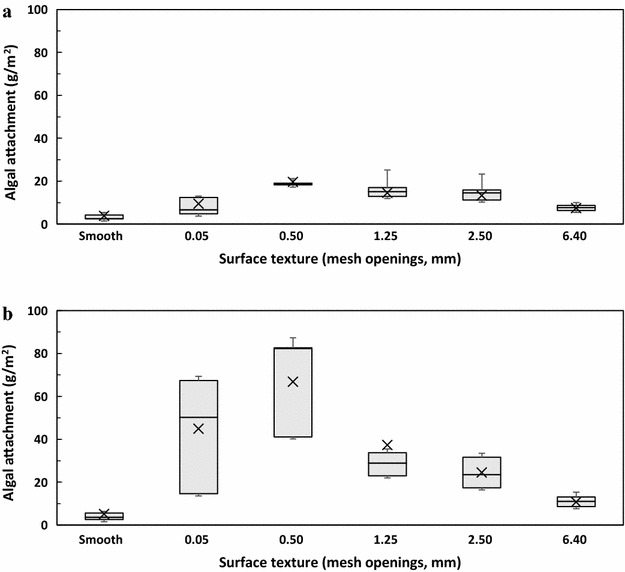
Algal attachment as a function of the material surface texture. **a** Stationary conditions and **b** rocking conditions. For each texture size, attachment on different materials (stainless steel, aluminum, polyester, HDPE, nylon, polypropylene) were grouped together to draw the box chart

**Fig. 5:**
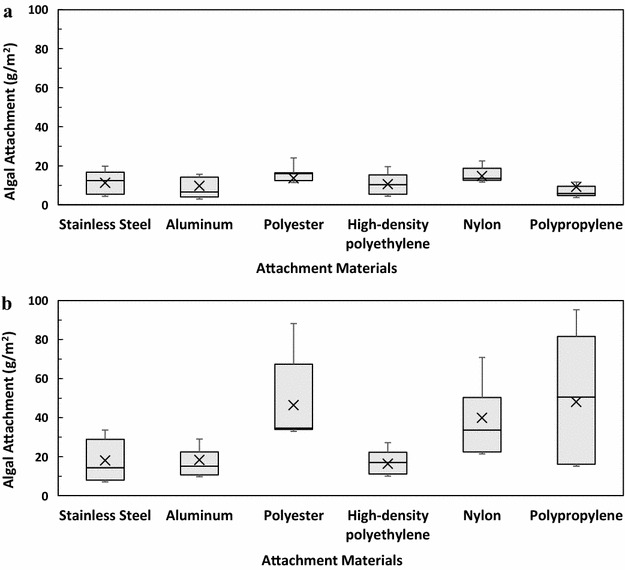
Algal attachment as a function of the materials. **a** Stationary conditions and **b** rocking conditions. For each material, attachment on different surface textures (smooth, 0.05, 0.5, 1.25, 2.50, 6.40 mm) were grouped together to draw the box chart

We first used a Tukey’s honestly significant difference (HSD) test to rank cell attachment among all of the 32 combinations of the materials and the textures. Under stationary conditions, 29 material–texture combinations showed no significant difference for cell attachment out of 32 combinations. Only the polypropylene with a 0.5-mm mesh opening showed significantly higher attachment than the stainless steel and high-density polyethylene with smooth surfaces (data not shown). For the rocking test, however, a wide range of cell attachment was observed with different materials and surface textures (Table 2). Among the 32 material–texture combinations, six combinations (nylon 0.50 mm mesh; polypropylene 1.25, 0.5, and 0.05 mm meshes; and polyester 0.05 and 0.5 mm meshes) led to significantly (*p* < 0.05) higher attachment than the other combinations (Table 2). To evaluate the individual contribution of materials and their surface texture on algal attachment, the cell attachment was further plotted as a function of surface texture (Fig. 4), or as a function of different materials (Fig. 5) using a box chart. Each box shown in Fig. 4 is the lumped data from the same texture but different materials; while each box shown in Fig. 5 is the lumped data from the same material but different textures. As shown in Fig. 4, the variation of attachment under stationary conditions (Fig. 4a) was less than that of the rocking condition (Fig. 4b). For both stationary and rocking tests, statistical analysis showed that the cell attachment at a mesh opening of 0.5 mm was significantly higher than that at smooth material and mesh opening of 6.4 mm (*p* < 0.05). For other mesh openings, however, it is difficult to compare and rank their cell attachment performance due to the large experimental errors occurred. This trend of cell attachment with the mesh openings was similar to that observed in Fig. 2. Figure 5 shows that the attachment material does not significantly (*p* > 0.05) affect algal attachment under stationary growth conditions (Fig. 5a). However, under rocking conditions algal attachment is drastically affected by the material used (Fig. 5b). Polyester, nylon, and polypropylene exhibited significantly higher (*p* > 0.05) attachment than stainless steel, aluminum, and HDPE (Fig. 5b). It should be noted that Figs. 4 and 5 once again demonstrated that the rocking conditions resulted in a higher cell attachment than the stationary conditions, which agrees with earlier results (Fig. 3).

**Table 2:** Tukey’s honestly significant difference (HSD) test of cell attachment as a function of the materials and their surface textures under rocking conditions

Ranking	Material	Surface texture (mesh opening, mm)	Attachment (g m^−2^)^a^	Groups^b^
1	Nylon	0.50	91.16 ± 10.13	A
2	Polypropylene	1.25	88.25 ± 18.37	A
3	Polyester	0.50	82.59 ± 26.38	AB
4	Polypropylene	0.50	82.34 ± 9.93	AB
5	Polypropylene	0.05	79.24 ± 0.97	AB
6	Polyester	0.05	67.37 ± 16.70	ABC
7	Nylon	0.05	50.28 ± 12.29	BCD
8	Aluminum	0.50	41.13 ± 11.45	CDE
9	Stainless steel	0.50	36.39 ± 9.49	CDEF
10	Polyester	2.50	34.64 ± 11.06	DCEFG
11	Polyester	1.25	33.96 ± 10.67	DEFG
12	Nylon	2.50	33.66 ± 16.45	DEFG
13	Stainless steel	1.25	33.20 ± 2.12	DEFG
14	High-density polyethylene	2.50	25.39 ± 7.12	DEFG
15	Aluminum	1.25	24.73 ± 14.03	DEFG
16	Nylon	1.25	22.43 ± 9.04	DEFG
17	Polypropylene	2.50	21.82 ± 3.98	DEFG
18	High-density polyethylene	0.50	21.24 ± 8.63	DEFG
19	Stainless steel	2.50	15.91 ± 10.45	EFG
20	Aluminum	2.50	15.54 ± 6.19	EFG
21	Aluminum	0.05	14.64 ± 1.91	EFG
22	Polypropylene	6.40	14.23 ± 4.75	EFG
23	Polyester	Smooth	13.19 ± 14.04	EFG
24	High-density polyethylene	6.40	12.77 ± 1.94	EFG
25	Stainless steel	0.05	12.72 ± 4.36	EFG
26	Aluminum	6.40	9.41 ± 2.10	EFG
27	Stainless steel	6.40	6.46 ± 3.67	FG
28	High-density polyethylene	Smooth	6.19 ± 5.19	FG
29	Aluminum	Smooth	4.07 ± 5.10	FG
30	Stainless steel	Smooth	3.27 ± 1.89	G
31	Polypropylene	Smooth	2.40 ± 0.83	G
32	Nylon	Smooth	1.84 ± 1.15	G

^a^Data are mean ± SDs of three replicates

^b^Attachment with at least one common letter are not significantly (*p* > 0.05) different

We further attempted to describe a quantitative relationship of algal attachment as a function of the materials and the surface texture. Here, the tetradecane contact angle $(\theta_{\text{te}} )$ was used to quantitatively represent the material surface physico-chemical properties because of its high correlation to algal attachment (*R*^2^ = 0.68). We also introduce Wenzel’s number (*r*) as the quantitative representation of the surface texture [19], i.e.,

1 $$ r = \frac{a}{A} $$

where *a* is the actual surface area of the material of a rough surface and *A* is the geometry of the projected area, i.e., the incidence area of a light perpendicular to the textured surface. A second-order polynomial model was used to correlate algal attachment with $\theta_{\text{te}}$ and *r* based on the experimental data obtained in Table 2. It should be noted that the data with *r* < 1.60 and those with *r* ≥ 1.60 were respectively correlated in order to gain a better correlation. Table 3 lists the estimates of the coefficients and associated *p* values obtained from the second-order polynomial regression.

**Table 3:** Estimates of coefficients of the variables in the second-order polynomial regression with different Wenzel’s numbers

Coefficient	Variables	*r* < 1.60		*r* ≥ 1.60	
		Estimate	*p* value	Estimate	*p* value
$\alpha_{0}$	Constant	156.2	0.08	104.6	0.15
$\alpha_{1}$	$\theta_{\text{te}}$	−2.2	0.43	19.4	<0.05
$\alpha_{2}$	*r*	275.3	<0.05	65.4	0.13
$\alpha_{12}$	$\theta_{\text{te}} \times r$	3.7	<0.05	1.7	0.69
$\alpha_{11}$	$\theta_{\text{te}}^{2}$	−0.1	0.38	0.7	<0.05
$\alpha_{22}$	*r* ^2^	122.6	<0.05	n.a.^a^	n.a.
Regression coefficient (*R* ^2^)		0.57		0.75	
*p* value		<0.001		<0.001	

The equation $Y = \alpha_{0} + \alpha_{1} \theta_{\text{te}} + \alpha_{2} r + \alpha_{12} \theta_{\text{te}} r + \alpha_{11} \theta_{\text{te}}^{2} + \alpha_{22} r^{2}$ was used for correlation, where *Y* is cell attachment (g m^−2^), *r* is the Wenzel’ number (dimensionless), and $\theta_{\text{te}}$ is tetradecane contact angle (°)

^a^The estimate was not available

**Fig. 6:**
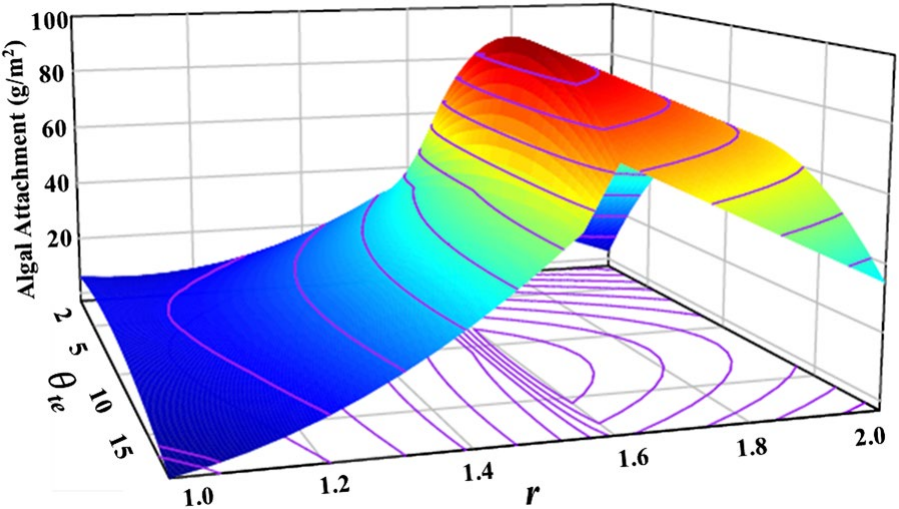
Response surface model of cell attachment as a function of material surface properties (tetradecane contact angle, $\theta_{\text{te}}$) and surface texture (Wenzel’s number, *r*)

A 3-D response surface (Fig. 6) was plotted based on the coefficients for the second polynomial model given in Table 3. As shown in the figure, with an increase in Wenzel’s number the algal attachment increases until it reaches 1.5–1.6 (corresponding to 0.5 mm mesh size). The change in cell attachment with tetradecane contact angles, however, was not as significant as the change in the Wenzel’s numbers. The optimal tetradecane contact angle varying with the Wenzel’s numbers indicates an interaction between the material and the surface texture.

### Long-term cell attached growth on different materials

**Fig. 7:**
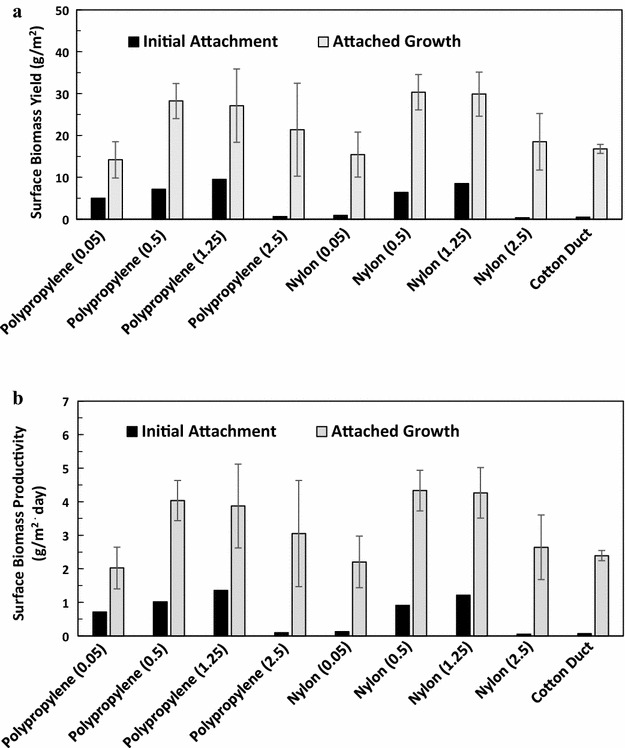
Algal attached growth on materials with various mesh openings. **a** Biomass yield per surface area of attachment materials and **b** biomass productivity (biomass yield divided by 7 days) per surface area of attachment. Cells were incubated using a RAB system for 7 days for initial attachment (i.e., the first growth/harvest cycle), and then repeatedly harvested and re-grown for another five cycles at 7 days/cycle with a total of 35 days attached growth. The *figure in the parenthesis* is the mesh opening size, mm. The attached growth data are the average of five consecutive cycles and the errors show the standard deviations

Following the laboratory-scale testing, the materials exhibiting the best attachment (nylon and polypropylene with various surface textures) were further tested on a pilot-scale RAB system to evaluate long-term cell attached growth as a function of different materials. Cells were incubated on a RAB system for the first 7 days for initial attachment, and then subjected to five cycles of repeated harvesting and re-growth at 7 days/cycle for a total of 35 days of attached growth. As shown in Fig. 7a, the initial cell attachment on the materials was lower than the biomass yield during the attached growth stage. However, the overall biomass yield for the attached growth stage was correlated with the initial attachment biomass yield for each material. Figure 7b shows that biomass productivity has the same trend as biomass yield. The optimal surface textures for the attached growth stage were 0.5–1.25 mm openings for both the nylon and polypropylene, which was similar to the optimal surface texture for the initial attachment stage (Fig. 2).

We also used cotton duct material as the control attachment material as this material resulted in good attachment in our previous studies [21]. It was found that nylon and polypropylene sheets with 0.5, 1.25, and 2.5 mm openings showed better algal attached growth performance than cotton duct. Collectively, Fig. 7 shows that nylon and polypropylene with a 0.5–1.25 mm mesh opening were the best material–texture combination for the attached growth of algae, with a yield of approximately 29 g m^−2^ and a productivity of 4.2 g m^−2^ day^−1^ which is 73 % higher than previously reported for cotton duct (Fig. 7).

## Discussion

The mechanisms for cell attachment have been studied in algal biofilm systems. The surfaces physico-chemical properties of the materials have been reported to play significant roles in cell attachment. For example, Genin et al. [13] found that polar surface energy had a correlation (*R*^2^ = 0.69) with algal attachment based on a consortium of freshwater algae and six materials. Ozkan and Berberoglu [12] reported that acid–base interactions are the dominant mechanism for algal attachment and hydrophobic algae tend to form biofilms better than hydrophilic algae. Other factors such as hydrophobic interactions [16] and dispersive surface energy [15] have also been reported as affecting algal attachment.

In this work, we used free surface energy and contact angles as the parameters to represent the materials physico-chemical surface properties and their implication on cell attachment. To evaluate the effect of materials surface physico-chemical properties without the interference of surface texture, we first used the material with smooth surface for the cell attachment study. Our results indicate poor correlations of cell attachment with the surface energy, water contact angle, and glycerol contact angle. However, a reasonable correlation (*R*^2^ = 0.68) was found between cell attachment and tetradecane contact angle, indicating tetradecane contact angle may be an appropriate parameter to predict cell attachment. The results reported here are somewhat different from a previous report that surface energy [13] has good correlation with cell attachment. The reason may be due to the differing culture conditions and algal species used. Also, a very comprehensive group of materials was tested in our work, while only a limited number of materials were used in the previous study.

In addition to the surface physico-chemical properties, material surface texture also affected the algal attachment. Previous research has shown that algal attachment increased with increased surface texture. For example, Sekar et al. [16] observed an enhanced algal attachment on metals that had been sanded with different grits of sand paper. Cao et al. [18] created a dimpled surface of steel materials (6–8 µm in diameter and 2–3 µm in depth) which resulted in higher cell attachment than a smooth surface. Sathananthan et al. [22] reported that a V-shape groove pattern with the same size scale as the algal cells resulted in higher biomass productivity than the smooth materials. Cui et al. [19] studied the effect of three different patterns (ridge, pillar, and groove) on cell attachment and concluded that attachment was preferred when the pattern size was close to the cell diameter.

In this work, we altered the surface texture by attaching a mesh sheet to a smooth surface of the material; the resulting square pore texture significantly increased cell attachment. However, the trend of cell attachment with pore size observed in this work is different from that reported previously [19]. For example, our results indicate that a mesh opening of 0.5 mm is optimal for algal attachment; the size less than this value (e.g., 0.05 mm opening) exhibited lower attachment. On the contrary, Cui et al. [19] reported that algal attachment increased with decreasing pore size until the opening was equal to or smaller than the algal cells. Based on this conclusion, the 0.05-mm opening, which is larger than the cell size used (*Chlorella* sp. 10–20 µm), should have given higher cell attachment than the 0.5-mm opening. The reason for this difference is that the algae in our study appeared to form flocks, which were not easily “accommodated” by the 0.05-mm openings, while the Cui et al. [19] study used single cells. The SEM imaging also confirmed this hypothesis, i.e., the cells formed flocks during growth which would not easily fit into the 0.05 mesh opening size. The impact of algal cell flocking in biofilm systems needs to be carefully considered in future attachment materials development.

It should be noted that previous research on algal attachment has been done under either stationary liquid conditions [15, 19] or flowing liquid conditions [13, 22]. In this study, to provide the same baseline comparison, both stationary and flowing (rocking) conditions were used to investigate cell attachment. Our results indicate that cell attachment in stationary conditions was quite different from that obtained in rocking conditions. This may be due to the shear force applied to the algal biofilm under the rocking conditions as compared to the stationary condition which does not generate shear force. As liquid flow always exists in algal biofilm systems, we believe the rocking condition is more appropriate to mimic the true conditions in algal biofilm cultures. Therefore, future materials development should be investigated in rocking or similar systems with liquid flow instead of stationary conditions.

The above discussion shows that both surface physico-chemical properties and surface texture of the materials play important roles in algal attachment. In general, surface physico-chemical properties determine the thermodynamics of cell attachment, i.e., whether the cell can attach to the materials surface. The surface texture, however, determines the local hydrodynamic conditions the algal biofilm encounters, i.e., whether the attached algal cells can be “sheltered” from the shear stress and avoid sloughing off of the material surface. The increased surface area provided by the texture may be another mechanism for the better attachment performance of the textured materials. The ultimate algal attachment performance is the outcome of the interaction of the surface physico-chemical properties and surface texture. However, studies on the combined effects of these two group parameters on cell attachment are still very rare; it was also unclear what the relative importance of these two parameters is for cell attachment. To fill these gaps, we performed a thorough investigation of the interaction of these two groups of parameters and their roles on cell attachment. In particular, this co-effect was quantified with a second-order polynomial.

Another issue worth noting is the difference between the initial cell attachment to fresh material and cell attached growth, once the colony is established. The former is a usually a rapid process while the latter needs to be investigated in long-term cultivation. In the previous studies, these two concepts were not clearly defined and were intermingled. In this work, the research performed in first three “Results” sections focused on initial cell attachment, while the research in last Result section focused on long-term attached growth. The results show that similar to the initial cell attachment test, the cell attached growth also depends on both the material surface physico-chemical properties and the surface textures.

The pilot-scale attached growth experiments demonstrate that materials with an appropriate combination of surface physico-chemical properties and surface texture can lead to not only good short-term initial cell attachment, but also superior long-term attached growth. The optimal material/texture combinations were nylon and polypropylene mesh sheet with 0.5–1.25 mm openings. In our previous research, we used cotton duct sheet as the attachment material. Although good attachment was reported with the cotton duct sheet, this material tended to deteriorate after soaking in the liquid for 2–3 months [9]. On the contrary, the nylon- and polypropylene-based materials with an appropriate surface texture are economical and resistant to degradation, and therefore, more applicable for commercial implementation.

## Conclusions

This research reports a comprehensive study of the effects of material surface physico-chemical properties and texture on the initial colonization and the long-term attached growth in algal biofilm systems. The two properties and their interactions play important roles in both the initial colonization and sustained attached growth. The tetradecane contact angle and the Wenzel’s number for the materials were good parameters to correlate algal attachment. Collectively, it was found that polypropylene and nylon mesh with 0.5–1.25 mm openings were the best materials for initial cell attachment and long-term attached growth, with a biomass yield of 31 g m^−2^ and a productivity of 4.5 g m^−2^ day^−1^ achieved in a pilot-scale RAB system.

## Methods

### Algal strain and subculture

The microalgal cultures were taken from a raceway pond (2000 L) in the Algal Production Facility at the Iowa State University BioCentury Research Farm in Boone, IA, USA. The culture conditions for the open pond were described previously [8]. In short, the pond liquid surface was exposed to atmosphere CO_2_ level with natural local sunlight. The yearly light intensity change was reported previously [8]. The pond was initially inoculated with *Chlorella vulgaris* (UTEX #265) and has been operated for a year to establish a stable algal community. Over a yearlong continuous operation, the algal community in the pond has migrated from a pure *Chlorella* culture to a mixed/non-sterile culture with various green algae and cyanobacteria species. The algae culture collected from the raceway pond was then maintained in a flat panel reactor (16-L) prior to use as the seed for the attachment experiment. The flat panel rector was illuminated under natural sunlight and the temperature was maintained between 20 and 25 °C. The medium used for culture maintenance was Bolds Basal Medium.

### Algal cell attachment on different materials

A total of 28 materials with a smooth surface were tested for cell attachment (Table 1). The materials can be categorized as metal, plastic, and rubber. The selection of the materials was based on their nature of being readily available, non- or slowly degradable, and low cost. Each type of material was cut into three replicate square pieces (10 cm × 10 cm) and attached to the bottom of transparent Plexiglas chambers. Each chamber had a dimension of 70 cm × 25 cm × 20 cm, and thus could handle 12 pieces of materials at one time. The material pieces were randomly placed in different locations of the chamber. Three liters of algal culture with a cell density of 1 g L^−1^ was added to each chamber. The chambers were incubated at 20 °C under atmospheric CO_2_ conditions with continuous 110–120 µmol s^−1^ m^−2^ illumination using florescent lights for 7 days. The cell attachment was tested under either stationary or rocking conditions. To create a rocking motion, the chambers were placed on a rocking shaker with smooth gentle rocking at 15° from the horizontal plane at 20 cycles min^−1^.

To determine cell colonization on the materials, the chamber was lifted vertically to remove the settled, but unattached, algae from the material. The attachment materials were removed from the chamber. The cell colonization on each different material was evaluated with a Likert scale to determine the percentage of attached cells on the materials surface [23]. For each material, three trained researchers independently determined the percentage of colonization by visual observation and these were then averaged.

Six materials based on the criteria described earlier (“Results” section) were selected for further study to determine the effects of surface texture on cell attachment. To create different surface textures, commercially available mesh sheets with different pore sizes (0.05, 0.5, 1.25, 2.5, and 6.4 mm openings) were adhered to the same material with a smooth surface. The polyester sheet with 6.4 mm opening; high-density polyethylene sheet with 0.05 and 0.5 mm openings; and nylon sheet with 6.4 mm opening were not available. The mesh sheets were cut into 25 cm × 25 cm squares and attached to the bottom of the chambers, and incubated at 20 °C under atmospheric CO_2_ conditions with continuous 110–120 µmol s^−1^ m^−2^ of illumination for 7 days. The tests were performed under stationary and rocking conditions. Cell attachment on the materials with different textures was quantified by scrapping the biomass and measuring the cell dry weight.

### Evaluation of material surface physico-chemical properties

Sessile drop tests were used to determine the liquid contact angle of the materials [24]. In short, 5 µL of three reference liquids, distilled water, glycerol, and tetradecane were pipetted onto the surface of the material and a picture was taken using a Nikon D800 camera with an AF-S Nikkor 35 mm F/1.4G lens. The images were analyzed using imageJ and a Java plug-in, DropSnake 2.1. This software used a global model of a reference drop and obtained contact angles reflecting the whole drop profile.

The contact angles were then used to determine the surface energies of the materials using Young’s equation, i.e.,

2 $$ \gamma_{\text{S}} = \gamma_{\text{L}} \cos \theta + \gamma_{\text{SL}} $$

where $\gamma_{\text{S}}$ is the surface free energy of the solid material, $\gamma_{\text{L}}$ is the surface energy of the liquid, *θ* is the contact angle, and $\gamma_{\text{SL}}$ is the interfacial energy between solid and liquid. A Van Oss–Chaudhury–Good thermodynamic approach [25] was used to determine $\gamma_{\text{SL}} .$ In brief, $\gamma_{\text{SL}}$ consists of polar $(\gamma_{\text{SL}}^{\text{P}} )$ and non-polar $(\gamma_{\text{SL}}^{\text{LW}} )$ components, i.e.,

3 $$ \gamma_{\text{SL}} = \gamma_{\text{SL}}^{\text{P}} + \gamma_{\text{SL}}^{\text{LW}}. $$

The values of $\gamma_{\text{SL}}^{\text{P}}$ and $\gamma_{\text{SL}}^{\text{LW}}$ can be calculated as [25],

4 $$ \gamma_{\text{SL}}^{\text{P}} = 2\left( {\sqrt {\gamma_{\text{S}}^{ + } } - \sqrt {\gamma_{\text{L}}^{ + } } } \right) \cdot \left( {\sqrt {\gamma_{\text{S}}^{ - } } - \sqrt {\gamma_{\text{L}}^{ - } } } \right) $$

5 $$ \gamma_{\text{SL}}^{\text{LW}} = \left( {\sqrt {\gamma_{\text{SL}}^{\text{LW}} } - \sqrt {\gamma_{\text{L}}^{\text{LW}} } } \right)^{2} $$

where $$ \gamma_{\text{S}}^{ + } $$ and $$ \gamma_{\text{S}}^{ - } $$ are the acid and base interactions of the solid, $$ \gamma_{\text{L}}^{ + } $$ and $$ \gamma_{\text{L}}^{ - } $$ are the acid and base interactions of the liquid, and $$ \gamma_{\text{S}}^{\text{LW}} $$ and $$ \gamma_{\text{L}}^{\text{LW}} $$ are Lifshitz-van der Waals forces/interactions for solid and liquid, respectively. The solid properties, $$ \gamma_{\text{S}}^{ + } , $$$$ \gamma_{\text{S}}^{ - } , $$ and $$ \gamma_{\text{S}}^{\text{LW}} , $$ can be obtained through the van Oss–Chaudhury–Good equation, i.e.,

6 $$ (1 + \cos \theta )\gamma_{\text{L}} = 2\left( {\sqrt {\gamma_{\text{S}}^{\text{LW}} \gamma_{\text{L}}^{\text{LW}} } + \sqrt {\gamma_{\text{S}}^{ + } \gamma_{\text{L}}^{ - } } + \sqrt {\gamma_{\text{S}}^{ + } \gamma_{\text{L}}^{ - } } } \right). $$

As the values of $$ \gamma_{\text{L}}^{ + } , $$$$ \gamma_{\text{L}}^{ - } , $$ and $$ \gamma_{\text{L}}^{\text{LW}} $$ can be known through choosing an appropriate liquid, the unknown variables from Eq. (6) are $$ \gamma_{\text{S}}^{ + } , $$$$ \gamma_{\text{S}}^{ - } , $$ and $$ \gamma_{\text{S}}^{\text{LW}} . $$ By applying a non-polar liquid (tetradecane) to Eq. (6), the equation becomes,

7 $$ (1 + \cos \theta )\gamma_{\text{L}} = 2\left( {\sqrt {\gamma_{\text{S}}^{\text{LW}} \gamma_{\text{L}}^{\text{LW}} } } \right) $$

From which the value of $\gamma_{\text{S}}^{\text{LW}}$ can be obtained. Then, applying two other polar liquids (water and glycerol) and Eq. (6) twice, the two remaining unknowns, $\gamma_{\text{S}}^{ + }$ and $\gamma_{\text{S}}^{ - } ,$ can be solved.

### Scanning electron microscopy

A Quanta FEG 250 SEM was used to image the algal attachment on the mesh materials in E-SEM (environmental SEM) mode. The chamber was set at 3 Torr of water vapor pressure to try to minimize drying. The SEM was operated at 20 kV accelerating voltage with a working distance of 14 mm. FEI’s gaseous secondary electron detector (GSED) was used to collect a secondary electron image.

### Cell attached growth on different materials at pilot-scale

Polypropylene and nylon demonstrated the best algal attachment and were selected for further testing to determine their performance for long-term algal attached growth using a pilot-scale RAB system. The detailed design and operation of the RAB system was described in our previous publication [8]. In short, RAB system was placed in the same environment as the open pond at the Iowa State University BioCentury Research Farm in Boone, IA, USA. The RAB system consisted of vertical conveyor belts rotating though a standard raceway pond at a linear velocity of 4 cm s^−1^. The algal biofilm on the belt was exposed to atmosphere CO_2_ level with natural local sunlight [8]. For each material, four different levels of surface texture (0.05, 0.5, 1.25, and 2.5 mm mesh size) were used to support the attached algal growth for a total of 42 days of operation. During this period, the cells were harvested every 7 days; thus, there was a total of 6 harvest/re-growth cycles. During each harvest, attached cells were scraped from 1 ft^2^ of the individual attachment material and then freeze-dried to identify the dry weight.

### Statistical analysis

All the tests were performed in triplicate, with the results being presented as the mean ± SD or as a box chart. Three-way analysis of variance (ANOVA) was used to test whether a significant difference exists among all of the groups of data. If there was a significance difference among the groups of the data, a Tukey’s HSD test was further performed to identify whether a specific group of data was significantly different from another. The software R [26] was used to perform ANOVA and HSD. The statistics related with the regression of cell attachment versus contact angle and surface roughness was also conducted using the software R [26].

## Abbreviations

HSD: Tukey’s honestly significant difference
RAB: revolving algal biofilm
SEM: scanning electron microscopy
*θ*: contact angle
*θ*_te_: tetradecane contact angle
*r*: Wenzel’s number
$\gamma_{\text{S}}$: surface free energy of the solid material
$\gamma_{\text{L}}$: surface energy of the liquid
$\gamma_{\text{SL}}$: interfacial energy between solid and liquid
$\gamma_{\text{SL}}^{\text{P}}$: polar components of $\gamma_{\text{SL}}$
$\gamma_{\text{SL}}^{\text{LW}}$: non-polar of components of $\gamma_{\text{SL}}$
$\gamma_{\text{S}}^{ + }$ and $\gamma_{\text{S}}^{ - }$: acid and base interactions of the solid, respectively
$\gamma_{\text{L}}^{ + }$ and $\gamma_{\text{L}}^{ - }$: acid and base interactions of the liquid, respectively
$\gamma_{\text{S}}^{\text{LW}}$ and $\gamma_{\text{L}}^{\text{LW}}$: Lifshitz-van der Waals forces/interactions for solid and liquid, respectively
